# Body mass index and peripheral neuropathy across the glucose tolerance spectrum in Japanese adults

**DOI:** 10.1111/jdi.70319

**Published:** 2026-05-13

**Authors:** Fukashi Ishibashi, Aiko Kosaka, Harumi Uetake, Darren C. Greenwood, Laura Güdemann, Hassan Fadavi, Mitra Tavakoli

**Affiliations:** ^1^ Internal Medicine Ishibashi Medical and Diabetes Centre Hiroshima Japan; ^2^ Leeds Institute for Data Analytics, School of Medicine University of Leeds Leeds UK; ^3^ Exeter Diabetes Centre of Excellence (EXCEED), Diabetes and Vascular Research Centre, National Institute for Health Research (NIHR), Exeter Clinical Research Facility University of Exeter Medical School Exeter UK

**Keywords:** Diabetes, Obesity, Peripheral neuropathy

## Abstract

**Aims:**

To examine the association between obesity, defined using population‐specific body mass index (BMI) thresholds, and peripheral neuropathy across the glucose tolerance spectrum in Japanese adults.

**Methods:**

This cross‐sectional study included 353 Japanese adults: 90 with normal glucose tolerance (NGT), 55 with impaired glucose tolerance (IGT), and 208 with type 2 diabetes mellitus. Participants were stratified by BMI (<25 vs ≥25 kg/m^2^) according to Japan Society for the Study of Obesity criteria. Peripheral neuropathy was assessed using the Neuropathy Disability Score, nerve conduction studies, quantitative sensory testing, and corneal confocal microscopy (CCM). Associations between BMI and neuropathy measures were examined using regression models adjusted for age and sex.

**Results:**

BMI‐related differences in neuropathy measures were predominantly confined to participants with type 2 diabetes mellitus. Within this group, higher BMI was associated with greater neuropathy severity (adjusted *P* = 0.01) and a higher prevalence of Toronto‐defined neuropathy (adjusted odds ratio 3.3, 95% CI 1.4–7.7; *P* = 0.01). In contrast, obesity was not independently associated with neuropathy measures in NGT or IGT participants. Corneal nerve fiber length was significantly reduced in IGT compared with NGT (*P* < 0.001), indicating early small‐fiber involvement related to glycemic status rather than adiposity.

**Conclusions:**

In Japanese adults, obesity at population‐specific BMI thresholds exacerbates peripheral neuropathy primarily in established type 2 diabetes mellitus. Early small‐fiber abnormalities are detectable in IGT and appear driven by glucose dysregulation rather than obesity alone. These findings highlight population‐specific patterns of neuropathy risk and support early identification of nerve involvement across the glucose tolerance spectrum in East Asian populations.

## Graphical abstract

Graphical illustration of participant classification by metabolic status (normoglycemic, IGT, type 2 diabetes mellitus) and BMI with key neurological and metabolic assessments. In Japanese adults, obesity at the Japanese BMI threshold (≥25 kg/m^2^) is associated with adverse metabolic profiles across all glycemic states but exacerbates peripheral neuropathy predominantly in established type 2 diabetes. Early nerve abnormalities in impaired glucose tolerance are detectable using sensitive measures (including corneal confocal microscopy) and appear more closely related to glycemic dysregulation than adiposity alone.

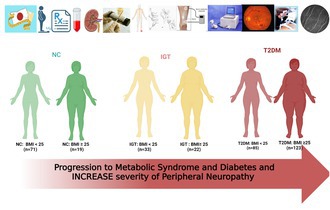

**Abbreviations**
ACR: albumin‐to‐creatinine ratio
BMI: body mass index
CCM: corneal confocal microscopy
CNBD: corneal nerve branch density
CNF: corneal nerve fiber
CNFL: corneal nerve fiber length
CPT: cold perception threshold
CV: coefficient of variation
CVR‐R: coefficient of variation of R‐R interval
DBP: diastolic blood pressure
eGFR: estimated glomerular filtration rate
HDL: high‐density lipoprotein cholesterol
IGT: impaired glucose tolerance
JASSO: Japan Society for the Study of Obesity
LDL: low‐density lipoprotein cholesterol
MCV: motor nerve conduction velocity
NCV: nerve conduction velocity
NDS: Neuropathy Disability Score
NGT: normoglycemic control
OGTT: oral glucose tolerance test
SBP: systolic blood pressure
SCV: sensory nerve conduction velocity
VPT: vibration perception threshold
WPT: warm perception threshold

## INTRODUCTION

Peripheral neuropathy (PN) is a common and debilitating complication of diabetes and metabolic disease, contributing to chronic pain, sensory loss, gait instability, foot ulceration, amputation, and premature mortality^1^. Although PN has traditionally been attributed to sustained hyperglycemia in type 2 diabetes mellitus, accumulating evidence indicates that excess adiposity itself plays a critical role in early nerve injury. Obesity is now recognized as one of the strongest risk factors for PN after diabetes^2^, and neuropathic changes have been reported in individuals with impaired glucose tolerance (IGT) and even normoglycemia, implicating obesity‐related metabolic and microvascular dysfunction in PN pathogenesis independent of established diabetes^2^, ^3^.

In Japan, PN is both prevalent and frequently underrecognized. Community‐based studies show that a substantial proportion of adults with diabetes experience painful diabetic neuropathy, which markedly impairs quality of life, yet physicians identify only a fraction of cases^4^. This occurs in the context of a high comorbidity burden: more than one‐third of Japanese adults with type 2 diabetes mellitus are overweight or obese (BMI ≥25 kg/m^2^), and many have additional chronic conditions such as cardiovascular disease, chronic kidney disease, or retinopathy^5^. Together, these data highlight the growing intersection of adiposity, metabolic disease, and neuropathy in this population.

The prevalence and magnitude of obesity–neuropathy associations differ substantially across populations. Western studies, using the World Health Organization definition of obesity (BMI ≥30 kg/m^2^), consistently report a strong link between obesity and PN^6^, ^7^, ^8^. In contrast, East Asian cohorts, including Japanese adults, develop glucose intolerance, central adiposity, and metabolic complications at lower BMI levels^8^, ^9^. The Japan Society for the Study of Obesity, therefore, defines obesity as BMI ≥25 kg/m^2^, further subdivided into severity grades, and international guidelines recommend lower BMI thresholds for East Asian populations (overweight ≥23.0 kg/m^2^; obesity ≥25.0 kg/m^2^)^10^. Failure to apply these population‐specific criteria may underestimate obesity‐related neuropathy risk, and differences in neuropathy assessment may further contribute to inconsistent findings. Interestingly, community‐based studies in Japan have reported little or no increase in clinically defined polyneuropathy among individuals with prediabetes or metabolic syndrome, even when BMI exceeds 25 kg/m^2^—a phenomenon sometimes termed the “Japanese paradox”^8^, ^11^, ^12^, ^13^, ^14^.

The present study aimed to determine whether obesity, defined using Japanese‐specific BMI thresholds, is independently associated with peripheral neuropathy across normoglycemia, prediabetes, and type 2 diabetes. By examining neuropathy risk across the full glucose tolerance spectrum, we sought to clarify the contribution of adiposity to peripheral nerve injury in an East Asian population and to inform population‐specific risk stratification.

## RESEARCH DESIGN AND METHODS

This cross‐sectional, observational study was conducted between November 2015 and August 2019 at the Ishibashi Medical Centre in Hiroshima, Japan. A total of 353 participants were enrolled, including 208 individuals with type 2 diabetes mellitus, 55 with IGT, and 90 normoglycemic control, with normal glucose tolerance (NGT) confirmed by oral glucose tolerance testing (OGTT).

Participants with IGT and type 2 diabetes mellitus were referred by health check organizations or other medical practitioners, although not specifically for diabetes management. Normoglycemic control participants attended the clinic for evaluation of non‐diabetic conditions and were confirmed to have NGT on OGTT.

Exclusion criteria, pre‐specified in the study protocol, included any clinically evident causes of peripheral neuropathy unrelated to diabetes, including hypothyroidism, vitamin B12 deficiency, or excessive alcohol consumption. Participants with corneal disease or dystrophy, prior refractive surgery, or current use of hard contact lenses were excluded to ensure the validity of corneal confocal microscopy (CCM) assessments.

The study protocol was approved by the ethics committee of the Ishibashi Medical Centre. Written informed consent was obtained from all participants. The study was conducted in accordance with the Declaration of Helsinki and adhered to the Strengthening the Reporting of Observational Studies in Epidemiology (STROBE) guidelines.

### Clinical and laboratory data

Comprehensive clinical, neurological, and ophthalmological assessments were performed in accordance with national guidelines and standard protocols at the Ishibashi Medical Centre. Measurements included body mass index (BMI), blood pressure, lipid profiles, glycated hemoglobin (HbA1c), and renal parameters, including estimated glomerular filtration rate (eGFR), urinary creatinine, and urinary albumin levels.

Fasting laboratory assessments were conducted following a 10–12 h fast, including a lipid panel. Normoglycemic control participants and those with IGT underwent a standard 75‐g oral glucose tolerance test. Diabetes and prediabetes (IGT and impaired fasting glucose) were defined according to American Diabetes Association criteria^15^.

Participants were stratified by BMI using Japanese‐specific thresholds into two categories: <25.0 kg/m^2^ and ≥25.0 kg/m^2^, with the latter classified as obese according to national and international recommendations and Japanese guidelines^8^, ^9^, ^13^, ^16^.

### Assessment of neuropathy and neurophysiological examinations

The severity of peripheral neuropathy and associated neurological deficits was assessed using the modified Neuropathy Disability Score (NDS), which includes evaluation of vibration perception, pinprick sensation, temperature perception, and ankle reflexes^17^. Neuropathy was defined using criteria consistent with the Toronto Consensus for probable/confirmed diabetic neuropathy, which requires the presence of abnormal nerve conduction in combination with at least one neuropathic symptom or sign^18^.

Accordingly, participants with NDS >2 and a reduced sensory conduction velocity (SCV) of the sural nerve were classified as having peripheral neuropathy. The threshold for abnormal sural nerve SCV was defined as <42 m/s, based on the mean minus two standard deviations (49.99 ± 8.02 m/s) derived from a reference cohort of 98 healthy control subjects matched for age, sex, and height to the type 2 diabetes mellitus group and assessed at the Ishibashi Medical Centre^19^.

To provide a comprehensive and standardized assessment of neuropathy severity, an average composite *z*‐score was calculated from eight neurophysiological parameters: median motor nerve conduction velocity and amplitude; sural sensory nerve conduction velocity and amplitude; vibration perception threshold; warm perception threshold; cold perception threshold; and the coefficient of variation of R‐R intervals (CVR‐R). This composite score enabled integration of large‐fiber, small‐fiber, and autonomic nerve function into a single quantitative measure of neuropathy severity.

All participants underwent detailed neurophysiological evaluations. Nerve conduction studies were performed using an electromyography system (Neuropak S1, Nihon Kohden, Tokyo, Japan). Motor nerve conduction velocity and compound muscle action potential amplitude were measured in the median nerve, while SCV and sensory nerve action potential amplitude were assessed in the ulnar and sural nerves. Skin temperature was maintained above 32°C during all electrophysiological assessments.

Vibration perception threshold was measured at the left medial malleolus using a biothesiometer (Biomedical Instruments, Newbury, OH, USA). Thermal perception thresholds, including warm and cold perception thresholds, were assessed at the dorsum of the foot using a thermal stimulator (Intercross‐200, Intercross Co., Tokyo, Japan). Cardiovagal autonomic function was evaluated by calculating the coefficient of variation of R‐R intervals (CVR‐R) from 200 consecutive electrocardiogram R‐R intervals^18^.

### Corneal confocal microscopy

All participants underwent *in vivo* CCM using the Heidelberg Retina Tomograph III equipped with the Rostock Corneal Module (HRT III–RCM; Heidelberg Engineering, Heidelberg, Germany) to image small corneal nerve fibers (CNFs) within the sub‐basal nerve plexus, in accordance with established protocol^20^, ^21^, ^22^. Six high‐quality, non‐overlapping images of the central cornea at the level of Bowman's layer were acquired from each eye.

Images were analyzed to quantify CNF morphology, including corneal nerve fiber density (CNFD), defined as the number of major nerve fibers per mm^2^ of corneal tissue; corneal nerve fiber length (CNFL), defined as the total length of all nerve fibers per mm^2^; and corneal nerve branch density (CNBD), defined as the number of branches arising from major nerve trunks per mm^2^. Image analysis was performed using ImageJ software (Texelcraft, Tokyo, Japan).

To minimize observer bias, image acquisition and analysis were performed by investigators blinded to participants' clinical and metabolic status throughout the study.

### Statistical methodology

Summary descriptive statistics are presented as means with 95% confidence intervals (CIs), stratified by diabetic status (i.e., type 2 diabetes mellitus, IGT and NGT) and BMI (obese and non‐obese). Geometric means with 95% CIs were used for log‐normal data, and median with bootstrapped 95% CI for ordinal data.

While no formal power calculation was conducted, the subsequent confidence intervals indicated that the achieved sample size was adequate to compare BMI groups and diabetic and metabolic statuses for most neurological markers with good precision.

Associations between neurological markers and obesity were estimated using linear regression, both unadjusted and adjusted for age and sex, with estimates interpreted as differences in means between obese compared to non‐obese. Neurological markers were log‐transformed where necessary, with estimates interpreted as the ratio of geometric means for obese to non‐obese.

Associations between neurological markers and diabetic status (type 2 diabetes mellitus, IGT and NGT) were modeled in a similar way with additional adjustment for BMI. Lipid markers were also modeled using the same methods.

Associations between neuropathy (binary) defined by the Toronto Consensus and obesity and diabetic status were estimated using logistic regression and presented as odds ratios, where the number of participants with the outcome permitted.

All statistical analyses were performed using Stata version 18. Two‐sided *P*‐values are used throughout, with *P* < 0.05 considered statistically significant.

## RESULTS

### Participant characteristics and metabolic profile

Table 1 summarizes the demographic and metabolic characteristics, while Table 2 presents the neurological characteristics of the study participants, stratified by glucose tolerance status (NGT, IGT, and type 2 diabetes mellitus) and BMI using Japanese‐specific thresholds (<25 and ≥ 25 kg/m^2^).

**Table 1:** Characteristics of study participants, by diabetic status/glucose tolerance test and BMI

	NGT			IGT (IGT, IGT + IFG)				Type 2 diabetes mellitus			
	BMI < 25 kg/m^2^	BMI ≥ 25 kg/m^2^	*P*‐value^a^	BMI < 25 kg/m^2^	BMI ≥ 25 kg/m^2^	*P*‐value^a^	*P*‐value^b^	BMI < 25 kg/m^2^	BMI ≥ 25 kg/m^2^	*P*‐value^a^	*P*‐value^b^
Numbers	*n* = 71	*n* = 19	–	*n* = 33 (32,1)	*n* = 22 (11, 11)	–	–	*n* = 85	*n* = 123	–	–
Male (%)	34 (48)	14 (74)	0.05	13 (40)	18 (82)	0.003	0.7	54 (64)	84 (68)	0.5	0.03
Mean age (years)	49.3 (47.1, 51.4)	53.8 (49.8, 57.8)	0.05	53.3 (48.5, 58.1)	47.1 (42.1, 52.2)	0.09	0.7	53.1 (51.4, 54.7)	48.8 (47.3, 50.3)	<0.001	0.8
Mean body mass index (kg/m^2^)	21.5 (20.9, 22.0)	27.2 (26.5, 27.8)	<0.001	20.8 (20.0, 21.7)	29.3 (27.4, 31.1)	<0.001	0.03	22.2 (21.9, 22.6)	30.2 (29.2, 31.1)	<0.001	<0.001
Median duration of diabetes (years)	–	–		–	–	–	–	6 (5, 8)	7 (6, 9)	0.4	–
Mean HbA1c (mmol/mol)	37.1 (36.3, 37.9)	39.0 (37.5, 40.6)	0.03	41.9 (40.9, 43.0)	42.1 (41.0, 43.2)	0.9	<0.001	63.6 (58.9, 68.4)	65.8 (62.2, 69.5)	0.5	<0.001
Mean eGFR (mL/min)	82.5 (78.6, 86.3)	77.5 (71.7, 83.2)	0.2	73.2 (69.0, 77.4)	80.1 (73.7, 86.6)	0.06	0.03	81.9 (77.4, 86.4)	85.1 (81.6, 88.6)	0.3	0.3
Median Urinary albumin to creatinine ratio (mg/gCr) (IQR)	6 (4, 10)	6 (4, 8)	0.9	6 (4, 10)	6 (4, 17)	0.5	0.6	8 (5, 14)	11 (6, 34)	0.08	<0.001
Mean high‐density lipoprotein (HDL) cholesterol (mmol/L)	1.9 (1.7, 2.0)	1.4 (1.3, 1.5)	<0.001	1.9 (1.7, 2.0)	1.4 (1.2, 1.5)	<0.001	0.2	1.6 (1.5, 1.7)	1.3 (1.3, 1.4)	<0.001	<0.001
Mean low‐density lipoprotein (LDL) cholesterol (mmol/L)	3.1 (2.9, 3.2)	3.3 (2.9, 3.6)	0.3	3.3 (3.1, 3.6)	3.6 (3.2, 4.0)	0.2	0.009	3.4 (3.2, 3.5)	3.6 (3.5, 3.8)	0.04	<0.001
Geometric mean triglycerides (mmol/L)	1.2 (0.9, 1.5)	2.1 (1.4, 2.7)	0.006	1.1 (0.9, 1.3)	2.1 (1.3, 3.0)	0.001	0.3	1.8 (1.5, 2.1)	2.6 (2.2, 3.1)	<0.001	<0.001
Mean total cholesterol (mmol/L)	5.5 (5.3, 5.7)	5.6 (5.1, 6.0)	0.6	5.7 (5.4, 6.0)	5.9 (5.4, 6.5)	0.4	0.07	5.8 (5.5, 6.1)	6.2 (6.0, 6.4)	0.03	<0.001
Statin use (%)	6 (8)	4 (21)	0.12	2 (6)	3 (14)	0.3	0.7	8 (9)	12 (10)	0.9	0.7

^a^ Unadjusted comparison of BMI groups within diabetic status group.

^b^ Unadjusted comparison of diabetic status group vs normoglycemic controls (NGT).

**Table 2:** Neuropathy and neurophysiological characteristics of participants stratified by glycemia status and BMI

	NGT			IGT (IGT, IGT + IFG)				Type 2 diabetes mellitus				
	BMI < 25 kg/m^2^	BMI ≥ 25 kg/m^2^	*P*‐value^a^	BMI < 25 kg/m^2^	BMI ≥ 25 kg/m^2^	*P*‐value^a^	*P*‐value^b^	BMI < 25 kg/m^2^	BMI ≥ 25 kg/m^2^	*P*‐value^a^	*P*‐value^b^	
Median Neuropathy Disability Score (NDS) (0–10)	0 (0, 1)	0 (0, 1)	0.4	1 (1, 2)	2 (1, 3)	0.1	<0.001	3 (2, 4)	4 (3, 5)	0.02	<0.001	
Neuropathy defined by Toronto Consensus (%)	0 (0)	0 (0)	–	1 (3)	1 (5)	0.8	–	15 (18)	6 (5)	0.005	–	
Mean motor conduction velocity of median nerve (m/s)	57.7 (56.6, 58.8)	57.8 (55.1, 60.4)	1.0	55.8 (54.5, 57.2)	56.7 (55.2, 58.1)	0.4	0.04	53.4 (52.6, 54.2)	53.6 (52.8, 54.3)	0.8	<0.001	
Mean action potential amplitude of median nerve (μV)	8.9 (8.2, 9.7)	9.2 (8.1, 10.3)	0.8	8.2 (7.5, 8.9)	7.9 (6.6, 9.3)	0.7	0.06	7.2 (6.6, 7.9)	6.9 (6.4, 7.5)	0.5	<0.001	
Mean sensory conduction velocity of sural nerve (m/s)	48.9 (47.8, 50.0)	47.8 (46.0, 49.6)	0.3	45.8 (44.4, 47.2)	47.1 (44.9, 49.2)	0.3	0.003	45.9 (44.8, 46.9)	47.3 (46.5, 48.1)	0.03	0.001	
Mean action potential amplitude of sural nerve (μV)	16.2 (14.6, 17.7)	14.5 (11.2, 17.8)	0.3	12.0 (9.5, 14.4)	11.5 (9.1, 13.8)	0.8	<0.001	12.0 (10.7, 13.3)	11.3 (10.2, 12.3)	0.4	<0.001	
Geometric mean vibration perception threshold (μ/120c/s)	1.5 (1.3, 1.8)	1.9 (1.4, 2.6)	0.2	2.0 (1.5, 2.5)	2.5 (1.9, 3.1)	0.2	0.02	2.5 (2.1, 3.0)	2.6 (2.3, 3.0)	0.7	<0.001	
Mean coefficient of variation of R‐R intervals (%)	4.2 (3.9, 4.5)	3.8 (3.5, 4.1)	0.3	3.5 (3.0, 3.9)	3.8 (3.3, 4.3)	0.3	0.01	3.5 (3.2, 3.8)	3.3 (3.1, 3.5)	0.3	<0.001	
Mean warm perception threshold (W/m^2^)	−477 (−499, −454)	−482 (−524, −440)	0.8	−550 (−591, −510)	−607 (−678, −536)	0.1	<0.001	−571 (−604, −539)	−612 (−645, −580)	0.09	<0.001	
Mean cold perception threshold (W/m^2^)	466 (442, 490)	459 (411, 507)	0.8	524 (485, 564)	527 (484, 570)	0.9	0.001	510 (483, 537)	533 (509, 557)	0.2	<0.001	
Mean corneal nerve fiber density (CNFD) (no/mm^2^)	30.0 (28.6, 31.3)	29.4 (27.2, 31.6)	0.7	25.8 (24.3, 27.2)	23.9 (22.0, 25.7)	0.1	<0.001	23.9 (22.8, 24.9)	23.1 (22.2, 24.1)	0.3	<0.001	
Mean corneal nerve branch density (CNBD) (no/mm^2^)	12.2 (11.0, 13.3)	12.2 (9.9, 14.4)	1.0	10.8 (9.2, 12.5)	10.1 (8.1, 12.0)	0.5	0.04	11.0 (10.3, 11.7)	10.4 (9.7, 11.0)	0.2	0.002	
Mean corneal nerve fiber length (CNFL) (mm/mm^2^)	14.6 (14.0, 15.1)	14.3 (13.4, 15.2)	0.7	12.7 (12.0, 13.4)	12.2 (11.3, 13.1)	0.3	<0.001	12.2 (11.8, 12.7)	12.0 (11.6, 12.4)	0.4	<0.001	
Mean *z*‐score of 8 neurophysiological tests	0.46 (0.37, 0.55)	0.36 (0.21, 0.50)	0.5	−0.02 (−0.16, 0.13)	−0.01 (−0.15, 0.14)	0.7	<0.001	−0.15 (−0.27, −0.04)	−0.21 (−0.28, −0.14)	0.7	<0.001	

^a^ Unadjusted comparison of BMI groups within diabetic status group.

^b^ Unadjusted comparison of diabetic status group vs normoglycemic controls (NGT).

Across all glucose tolerance categories, participants with BMI ≥25 kg/m^2^ had significantly higher BMI values and a more adverse lipid profile, characterized by lower high‐density lipoprotein (HDL) cholesterol and higher triglyceride levels, compared with those with BMI <25 kg/m^2^. Age and sex distributions differed modestly between BMI strata within groups, while duration of diabetes did not differ significantly between BMI categories among participants with type 2 diabetes mellitus.

Glycemic control worsened progressively across NGT, IGT, and type 2 diabetes mellitus groups, as reflected by increasing HbA1c levels (*P* < 0.001), but did not differ substantially by BMI category within the same glucose tolerance group. Renal function, assessed by estimated eGFR and urinary albumin‐to‐creatinine ratio (UACR), showed no clinically meaningful differences between BMI strata in NGT or IGT participants, whereas higher albuminuria was observed in type 2 diabetes mellitus, particularly among individuals with BMI ≥25 kg/m^2^.

### Neuropathy and neurophysiological findings

Marked differences in neuropathy burden were observed across glucose tolerance groups (Table 2). Median NDS was 0 in NGT participants and 1 in those with IGT, but increased substantially in type 2 diabetes mellitus, with median scores of 3 in individuals with BMI <25 kg/m^2^ and 4 in those with BMI ≥25 kg/m^2^ (*P* < 0.001). Consistent with this, the prevalence of clinically defined neuropathy was 0% in NGT, 3% in IGT, and increased to 18% in participants with type 2 diabetes mellitus (*P* = 0.005).

Neurophysiological measures showed stepwise differences across glucose tolerance groups. Motor nerve conduction velocity and action potential amplitude were significantly reduced in type 2 diabetes mellitus compared with NGT and IGT, while sural SCV and amplitude were also lower in type 2 diabetes mellitus. Small‐fiber function showed similar patterns, with higher vibration perception thresholds and altered thermal perception thresholds in type 2 diabetes mellitus compared with NGT (all *P* < 0.001). Autonomic function, assessed by the coefficient of variation of R‐R intervals, was likewise reduced in type 2 diabetes mellitus.

When summarized using a composite *z*‐score derived from eight neurophysiological parameters, neuropathy severity was significantly greater in both IGT and type 2 diabetes mellitus compared with NGT participants (*P* < 0.001), indicating worsening neuropathy across the glucose tolerance spectrum.

**Figure 1:**
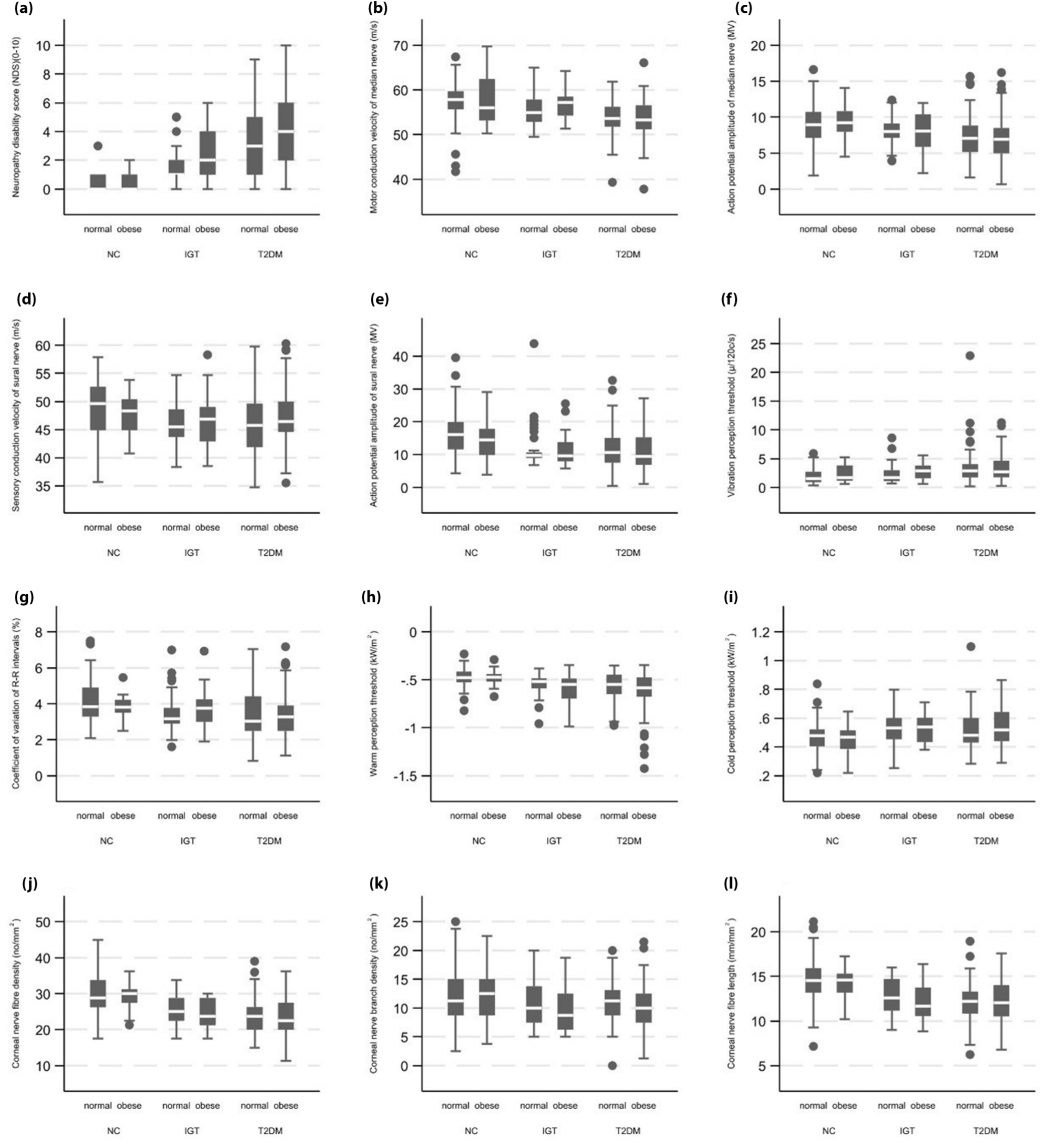
Boxplots of neurological and microvascular function in normal and obese individuals across normoglycemic controls (NCs), impaired glucose tolerance (IGT), and type 2 diabetes mellitus: (a) Neuropathy Disability Score (NDS), (b) median conduction velocity of the median nerve, (c) median sensory nerve action potential amplitude of the median nerve, (d) sural sensory nerve conduction velocity, (e) sural sensory nerve action potential amplitude, (f) vibration perception threshold, (g) coefficient of variation of R‐R intervals, (h) warm perception threshold, (i) cold perception threshold, (j) corneal nerve fiber density, (k)corneal nerve branch density, and (l) corneal nerve fiber length.

Figure 1 provides a visual illustration of the neurological markers in the table, unadjusted for confounding.

### BMI‐associated differences in metabolic, renal, and neurological measures

Table 3 presents differences in metabolic parameters, renal function, neurological measures, and CNF metrics between participants with BMI ≥25 kg/m^2^ and those with BMI <25 kg/m^2^ within each glucose tolerance group. Results are shown as unadjusted estimates and after adjustment for age and sex.

**Table 3:** Difference in mean lipid concentrations, kidney function, neurological markers, and small C‐nerve fiber markers by BMI and diabetic status

	NGT		IGT		Type 2 diabetes mellitus	
	BMI ≥ 25 vs < 25 kg/m^2^		BMI ≥ 25 vs < 25 kg/m^2^		BMI ≥ 25 vs < 25 kg/m^2^	
	Unadjusted	Adjusted^a^	Unadjusted	Adjusted^a^	Unadjusted	Adjusted^a^
High‐density lipoprotein (HDL) cholesterol (mmol/L)	−0.5 (−0.7, −0.2), *P* < 0.001	−0.4 (−0.6, −0.2), *P* < 0.001	−0.5 (−0.7, −0.3), *P* < 0.001	−0.4 (−0.6, −0.1), *P* = 0.002	−0.3 (−0.4, −0.2), *P* < 0.001	−0.2 (−0.3, −0.1), *P* < 0.001
Low‐density lipoprotein (LDL) cholesterol (mmol/L)	0.2 (−0.2, 0.5), *P* = 0.3	0.2 (−0.2, 0.6), *P* = 0.3	0.3 (−0.2, 0.7), *P* = 0.2	0.4 (−0.1, 0.9), *P* = 0.09	0.3 (0.0, 0.5), *P* = 0.04	0.3 (0.1, 0.6), *P* = 0.01
Triglycerides (mmol/L)^b^	1.9 (1.4, 2.6), *P* < 0.001	1.6 (1.1, 2.1), *P* = 0.006	1.7 (1.2, 2.2), *P* = 0.001	1.4 (1.0, 1.9), *P* = 0.05	1.5 (1.2, 1.8), *P* < 0.001	1.4 (1.2, 1.7), *P* < 0.001
Total cholesterol (mmol/L)	0.1 (−0.3, 0.6), *P* = 0.6	0.1 (−0.4, 0.6), *P* = 0.8	0.2 (−0.3, 0.8), *P* = 0.4	0.4 (−0.2, 1.0), *P* = 0.2	0.4 (0.0, 0.7), *P* = 0.03	0.4 (0.1, 0.8), *P* = 0.01
Ratio of total to HDL cholesterol^b^	1.3 (1.2, 1.5), *P* < 0.001	1.3 (1.1, 1.5), *P* = 0.001	1.4 (1.2, 1.6), *P* < 0.001	1.3 (1.1, 1.6), *P* < 0.001	1.3 (1.2, 1.4), *P* < 0.001	1.2 (1.1, 1.4), *P* < 0.001
Estimated glomerular filtration rate (eGFR) (mL/min/1.73m^2^)	−5 (−13, 3), *P* = 0.2	−2 (−9, 5), *P* = 0.5	7 (0, 14), *P* = 0.06	6 (−1, 13), *P* = 0.1	3 (−2, 9), *P* = 0.3	−1 (−6, 4), *P* = 0.7
Urinary albumin to creatinine ratio (ACR) (mg/g)^b^	1.0 (0.7, 1.5), *P* = 0.9	0.9 (0.6, 1.4), *P* = 0.6	1.2 (0.7, 2.2), *P* = 0.5	1.3 (0.7, 2.5), *P* = 0.4	1.4 (1.0, 1.9), *P* = 0.08	1.3 (0.9, 1.9), *P* = 0.1
Neuropathy Disability Score (NDS)	0.1 (−0.2, 0.5), *P* = 0. 4	0.2 (−0.1, 0.5), *P* = 0.3	0.7 (−0.2, 1.6), *P* = 0.1	0.4 (−0.5, 1.3), *P* = 0.4	0.9 (0.1, 1.6), *P* = 0.02	0.9 (0.2, 1.7), *P* = 0.01
Neuropathy defined by Toronto Consensus (Odds ratio)	‐	‐	‐	‐	5.0 (1.7, 10.0), *P* = 0.005	3.3 (1.4, 7.7), *P* = 0.01
Action potential amplitude of median nerve (μV)	0.2 (−1.3, 1.7), *P* = 0.8	0.0 (−1.6, 1.6), *P* = 1.0	−0.3 (−1.7, 1.1), *P* = 0.7	−0.5 (−2.1, 1.1), *P* = 0.5	−0.3 (−1.2, 0.5), *P* = 0.5	−0.4 (−1.3, 0.5), *P* = 0.3
Sensory conduction velocity of sural nerve (m/s)	−1.1 (−3.5, 1.2), *P* = 0.3	−0.9 (−3.4, 1.6), *P* = 0.5	1.2 (−1.2, 3.6), *P* = 0.3	1.0 (−1.7, 3.6), *P* = 0.5	1.5 (0.1, 2.8), *P* = 0.03	1.3 (−0.1, 2.6), *P* = 0.07
Action potential amplitude of sural nerve (μV)	−1.7 (−5.1, 1.7), *P* = 0.3	−2.3 (−5.8, 1.2), *P* = 0.2	−0.5 (−4.0, 3.0), *P* = 0.8	0.3 (−3.6, 4.2), *P* = 0.9	−0.7 (−2.4, 0.9), *P* = 0.4	−0.7 (−2.5, 1.0), *P* = 0.4
Vibration perception threshold (μ/120c/s)^b^	1.2 (0.9, 1.8), *P* = 0.2	1.1 (0.8, 1.7), *P* = 0.5	1.3 (0.9, 1.8), *P* = 0.2	1.5 (1.1, 2.2), *P* = 0.02	1.0 (0.9, 1.3), *P* = 0.7	1.1 (0.9, 1.4), *P* = 0.3
Coefficient of variation of R‐R intervals (%)	−0.3 (−1.0, 0.3), *P* = 0.3	−0.1 (−0.7, 0.5), *P* = 0.8	0.3 (−0.3, 0.9), *P* = 0.3	0.1 (−0.6, 0.8), *P* = 0.8	−0.2 (−0.5, 0.2), *P* = 0.3	−0.3 (−0.7, 0.0), *P* = 0.08
Warm perception threshold (W/m^2^)	−6 (−53, 42), *P* = 0.8	17 (−30, 65), *P* = 0.5	−56 (−131, 18), *P* = 0.1	−45 (−129, 39), *P* = 0.3	−41 (−88, 6), *P* = 0.09	−37 (−84, 10), *P* = 0.1
Cold perception threshold (W/m^2^)	−7 (−59, 45), *P* = 0.8	−21 (−75, 33), *P* = 0.4	3 (−56, 61), *P* = 0.9	−1 (−68, 66), *P* = 1.0	23 (−13, 59), *P* = 0.2	21 (−16, 58), *P* = 0.3
Corneal nerve fiber density (CNFD) (no/mm^2^)	−0.5 (−3.4, 2.3), *P* = 0.7	−1.4 (−4.3, 1.5), *P* = 0.3	−1.9 (−4.1, 0.4), *P* = 0.1	−2.4 (−4.9, 0.2), *P* = 0.07	−0.7 (−2.2, 0.7), *P* = 0.3	−0.6 (−2.1, 0.9), *P* = 0.5
Corneal nerve branch density (CNBD) (no/mm^2^)	0.0 (−2.5, 2.5), *P* = 1.0	0.5 (−2.1, 3.0), *P* = 0.7	−0.8 (−3.3, 1.7), *P* = 0.5	−0.2 (−3.0, 2.6), *P* = 0.9	−0.6 (−1.6, 0.3), *P* = 0.2	−0.8 (−1.8, 0.2), *P* = 0.1
Corneal nerve fiber length (CNFL) (mm/mm^2^)	−0.3 (−1.4, 0.9), *P* = 0.7	−0.4 (−1.6, 0.8), *P* = 0.5	−0.5 (−1.6, 0.5), *P* = 0.3	−0.6 (−1.8, 0.6), *P* = 0.3	−0.2 (−0.9, 0.4), *P* = 0.4	−0.2 (−0.9, 0.4), *P* = 0.5

*Note:* Odds ratios represent the odds of neuropathy in participants with BMI ≥ 25 kg/m^2^ compared with BMI < 25 kg/m^2^. Odds ratios were estimated only for the type 2 diabetes mellitus group due to absence or very low prevalence of neuropathy in NC and IGT.

^a^ Adjusted for age and sex.

^b^ Values represent ratios of geometric means.

Across all glucose tolerance categories, higher BMI was consistently associated with an adverse lipid profile. Participants with BMI ≥25 kg/m^2^ had significantly lower HDL cholesterol and higher triglyceride levels in NGT, IGT, and type 2 diabetes mellitus groups (all adjusted *P* ≤ 0.002). The ratio of total to HDL cholesterol was also significantly higher in obese participants across all glycemic groups (all adjusted *P* < 0.001). In contrast, low‐density lipoprotein cholesterol differed by BMI only in participants with type 2 diabetes mellitus, with higher levels observed in those with BMI ≥25 kg/m^2^ after adjustment (adjusted *P* = 0.01).

Renal function measures showed a limited association with BMI. eGFR and urinary albumin‐to‐creatinine ratio did not differ significantly between BMI categories in NGT or IGT participants. In type 2 diabetes mellitus, higher BMI was associated with a trend toward increased albuminuria, although this did not reach statistical significance after adjustment (adjusted *P* = 0.10).

BMI‐associated differences in neuropathy measures were evident predominantly in participants with type 2 diabetes mellitus. NDSs were significantly higher in type 2 diabetes mellitus participants with BMI ≥25 kg/m^2^ compared with those with BMI <25 kg/m^2^ (adjusted *P* = 0.01). Consistent with this, BMI ≥25 kg/m^2^ was associated with higher odds of clinically defined neuropathy according to the Toronto Consensus criteria (adjusted OR 3.3, 95% CI 1.4–7.7; *P* = 0.01; reference: BMI <25 kg/m^2^).

No significant BMI‐related differences in neuropathy measures were observed in NGT or IGT participants.

Electrophysiological measures showed limited BMI‐related differences. In participants with type 2 diabetes mellitus, sural SCV demonstrated a modest, non‐significant association with BMI after adjustment for age and sex (adjusted *P* = 0.07), indicating no independent effect of obesity on large‐fiber conduction velocity.

Increased vibration perception thresholds were observed in IGT participants with BMI ≥25 kg/m^2^ (*P* = 0.02), whereas no difference was evident in the type 2 diabetes mellitus group (*P* = 0.7).

Structural small‐fiber measures assessed by CCM, including CNFD, CNBD, and CNFL, did not differ significantly between BMI categories within any glucose tolerance group (all *P* > 0.05).

**Figure 2:**
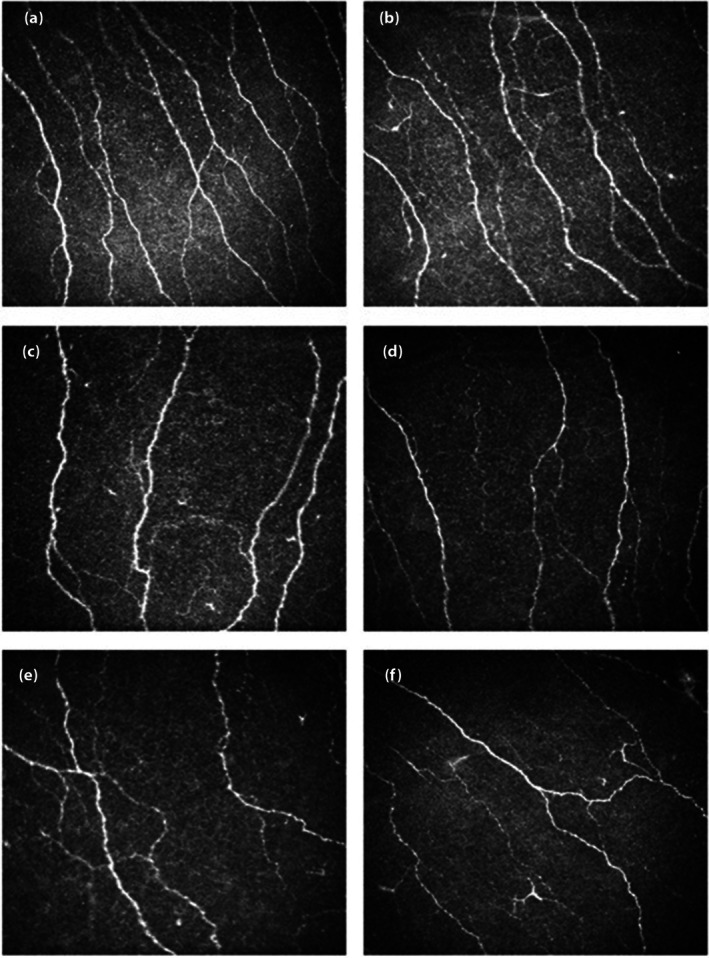
Representative of the corneal sub‐basal nerve plexus in normoglycemic control (NGT), impaired glucose tolerance (IGT), and type 2 diabetes mellitus subjects, with and without obesity. Images are shown for (a) an NGT subject without obesity, (b) an NGT subject with obesity, (c) an IGT subject without obesity, (d) an IGT subject with obesity, (e) a type 2 diabetes mellitus subject without obesity, and (f) a type 2 diabetes mellitus subject with obesity.

Figure 2 illustrates representative CCM images showing the corneal sub‐basal nerve plexus in individuals from different subgroups of the study. The images highlight variations in nerve fiber density and structure across NGT, IGT, and type 2 diabetes mellitus groups, stratified by BMI categories (<25 kg/m^2^ and ≥ 25 kg/m^2^).

### Neuropathy and corneal nerve measures across glucose tolerance groups

Compared with NGT, neuropathy severity differed across glucose tolerance groups. NDSs were significantly higher in both the IGT and type 2 diabetes mellitus groups relative to NGT (both *P* < 0.001). Large‐fiber neurophysiological assessment showed that median nerve action potential amplitude did not differ between IGT and NGT participants but was significantly reduced in the type 2 diabetes mellitus group (*P* < 0.001). In contrast, sural nerve SCV was significantly lower in both IGT (*P* = 0.003) and type 2 diabetes mellitus participants (*P* < 0.001) compared with NGT.

Sensory functional impairment was also evident, with vibration perception thresholds significantly elevated in both IGT (*P* = 0.02) and type 2 diabetes mellitus (*P* < 0.001) relative to NC. Structural small‐fiber abnormalities assessed by CCM were most pronounced in type 2 diabetes mellitus, where CNFD and CNBD were significantly reduced compared with NGT (both *P* < 0.001). CNFL was significantly reduced in both IGT and type 2 diabetes mellitus groups compared with NGT (both *P* < 0.001). These associations remained significant after adjustment for age, sex, and BMI.

## DISCUSSION

In this Japanese cohort spanning normoglycemia, IGT, and type 2 diabetes mellitus, we demonstrate three principal findings. First, neuropathy severity differed across glycemic status, with detectable functional and structural nerve abnormalities present in IGT and more advanced neuropathic involvement in type 2 diabetes mellitus. Second, obesity defined using Japanese‐specific criteria (BMI ≥25 kg/m^2^) was consistently associated with adverse metabolic profiles across all glycemic groups, but its impact on neuropathy measures was largely confined to individuals with established type 2 diabetes mellitus. Third, structural small‐fiber loss assessed by CCM was primarily related to glycemic status rather than adiposity, with no independent association between obesity and corneal nerve morphology in NGT or IGT participants. Together, these findings clarify population‐specific relationships between adiposity, glucose dysregulation, and peripheral neuropathy. They also highlight important differences in neuropathy pathophysiology within Japanese populations.

Obesity, even in the absence of abnormal glucose levels, has been shown to significantly increase the prevalence of neuropathy, as evidenced by intraepidermal nerve fiber density (IENFD) and nerve conduction studies (NCS)^23^. Obesity and IGT are pivotal metabolic factors intricately linked to peripheral nerve damage, with worsening glycemic status correlating with a higher prevalence of neuropathy, regardless of the criteria used to define the condition^2^. A study in Brazil reported that 11% of individuals with grade II and III obesity, metabolic syndrome (MetS), but without diabetes, had peripheral polyneuropathy, which was independently associated with low HDL levels^24^. In the PROMISE Cohort, prediabetes, rather than MetS, emerged as independently associated with both the presence of peripheral neuropathy and the severity of nerve dysfunction^25^.

### Neuropathy across the glucose tolerance spectrum in Japan

Our results demonstrate a clear gradient of neuropathy burden across glucose tolerance groups, with significant increases in NDSs, sensory conduction abnormalities, vibration perception thresholds, and CCM‐derived nerve alterations in type 2 diabetes mellitus, and intermediate abnormalities in IGT. These findings are consistent with our previous findings^19^, ^26^ and large Japanese cohorts, including the JDCP study in patients with type 2 diabetes mellitus^11^.

Importantly, our data extend prior Japanese population studies by showing that, although overt clinical polyneuropathy remains uncommon in IGT, measurable subclinical neuropathy is already present. This helps reconcile our findings with those of Kurisu *et al*., who reported that clinical polyneuropathy does not increase markedly in Japanese individuals with prediabetes or metabolic syndrome. Our results suggest that neuropathic changes in IGT occur at a stage below conventional clinical thresholds and may therefore be underestimated when assessment relies solely on symptoms or large‐fiber‐dominant criteria.

### Obesity, BMI thresholds, and the Japanese context

A central aim of this study was to determine whether obesity, defined using Japanese‐specific BMI thresholds, independently contributes to neuropathy risk across glycemic states. In line with JASSO guidance, obesity was associated with an adverse lipid profile across all glucose tolerance groups, reinforcing the metabolic relevance of BMI ≥25 kg/m^2^ in Japanese populations^8^.

However, obesity‐related differences in neuropathy measures were evident predominantly in individuals with established type 2 diabetes mellitus. Within this group, higher BMI was associated with significantly greater NDSs and a higher prevalence of clinically defined neuropathy, independent of age and sex. In contrast, obesity was not independently associated with neuropathy measures in normoglycemic or IGT participants.

Experimental and clinical studies indicate that hyperglycemia promotes oxidative stress, advanced glycation end‐product formation, mitochondrial dysfunction, and microvascular injury within peripheral nerves. When combined with obesity‐related metabolic disturbances‐ including systemic inflammation, dyslipidemia, adipokine imbalance, and insulin resistance‐ these processes may act synergistically to accelerate nerve degeneration. In contrast, individuals with normoglycemia or IGT may not yet have accumulated sufficient metabolic or microvascular injury for obesity alone to translate into clinically detectable neuropathy. Another contributing factor may relate to the sensitivity of neuropathy phenotyping. Conventional clinical and electrophysiological measures predominantly detect large‐fiber dysfunction, whereas early metabolic neuropathy may initially involve small‐fiber abnormalities that are not captured by routine assessments. This may partly explain why obesity‐related neuropathy signals were not evident in normoglycemic or IGT participants despite adverse metabolic profiles.

These findings are concordant with population‐based data from Japan. In a community cohort, Kurisu *et al*. reported that clinically defined polyneuropathy did not increase in individuals with prediabetes or metabolic syndrome, even in the presence of Japanese‐defined obesity^14^. Conversely, registry data from the JDCP study demonstrate a high prevalence of distal symmetric sensorimotor polyneuropathy among Japanese adults with type 2 diabetes, supporting the notion that sustained hyperglycemia is a key determinant of clinically overt neuropathy in this population^11^.

Together, these observations provide a physiological explanation for the so‐called “Japanese paradox,” whereby obesity and metabolic syndrome alone do not appear sufficient to drive clinically manifest peripheral neuropathy in the absence of established diabetes, despite clear metabolic derangement at lower BMI thresholds.

### Structural vs functional neuropathy

The inclusion of CCM allowed differentiation between functional neuropathy and structural small‐fiber degeneration. While functional abnormalities were detectable in IGT, structural small‐fiber loss—particularly reductions in CNF density and branching—was most evident in type 2 diabetes mellitus and showed no independent association with obesity across glycemic groups. This dissociation suggests that chronic glycemic exposure, rather than adiposity alone, is the dominant determinant of established small‐fiber neuropathy in this population. Consistent with this, obesity was not independently associated with large‐fiber electrophysiological measures, including sural SCV, after adjustment for confounders.

Notably, CNFL was reduced in both IGT and type 2 diabetes mellitus, even when nerve density and branching were preserved in IGT. This pattern is consistent with a staged process of neuropathy development, in which early fiber shortening precedes overt fiber loss. These findings support the use of CCM as a sensitive biomarker for detecting early neuropathy in Japanese individuals at metabolic risk.

### Clinical implications within the JASSO 2022 framework

These findings align closely with the 2022 Japan Society for the Study of Obesity (JASSO) guidelines, which define obesity (BMI ≥25 kg/m^2^) as a disease entity when accompanied by obesity‐related complications^8^. In the present cohort, individuals meeting Japanese criteria for obesity consistently demonstrated adverse metabolic profiles, particularly dyslipidemia, fulfilling key components of “obesity disease” as defined by JASSO. However, neuropathy‐related complications were largely confined to individuals with established type 2 diabetes mellitus.

This pattern suggests that, in Japanese populations, obesity alone at JASSO‐defined BMI thresholds may be insufficient to precipitate clinically overt peripheral neuropathy in the absence of sustained hyperglycemia. Nevertheless, the presence of early functional and structural nerve abnormalities in individuals with IGT highlights a clinically relevant stage at which metabolic risk is elevated but neuropathy remains subclinical. These findings support early identification and management of obesity and glucose dysregulation in the prediabetic phase, before more advanced structural small‐fiber neuropathy—as detected by CCM—becomes established.

### Strengths and limitations

This study is among the largest to integrate CCM, detailed neurophysiological assessment, and comprehensive metabolic profiling in a Japanese cohort spanning the full glucose tolerance spectrum. Use of the 75‐g oral glucose tolerance test ensured accurate classification of normoglycemic and IGT groups, reducing misclassification inherent in studies relying solely on HbA1c.

Several limitations should be acknowledged. The cross‐sectional design precludes inference regarding temporal progression or causality, and it remains uncertain whether obese individuals with IGT will develop neuropathy more rapidly than their leaner counterparts. Direct measures of visceral adiposity, such as visceral fat area assessed by computed tomography, were not available. Data on antihypertensive therapy, retinopathy status, and antidiabetic medications were also not systematically collected, limiting further clinical stratification, particularly within the type 2 diabetes mellitus group. Although lipid profiles were consistent with a visceral adiposity phenotype, this remains indirect and should be interpreted with caution. Furthermore, circulating inflammatory or adiposity‐related biomarkers that may mediate the relationship between obesity and peripheral nerve injury were not measured.

### Future directions

Longitudinal studies are required to determine whether early small‐fiber abnormalities in IGT progress to clinically overt neuropathy. Incorporation of direct measures of visceral adiposity and inflammatory biomarkers (e.g., adipokines and cytokines) will be important to better define underlying mechanisms. In addition, interventional studies are needed to evaluate whether early metabolic optimization, including lifestyle modification and weight‐reducing therapies, can prevent or attenuate structural nerve damage detected by sensitive biomarkers such as CCM.

## CONCLUSIONS

In summary, this study delineates population‐specific relationships between obesity, glycemic status, and peripheral neuropathy in a Japanese cohort. Neuropathy severity differs across the glucose tolerance spectrum, with early functional abnormalities detectable in IGT and more advanced neuropathic involvement in type 2 diabetes mellitus. Obesity at Japanese‐defined BMI thresholds contributes to adverse metabolic profiles across all groups but exacerbates neuropathy primarily in established diabetes. Structural small‐fiber neuropathy assessed by CCM appears associated predominantly with glycemic burden rather than adiposity alone. These findings support population‐specific neuropathy screening strategies and early intervention targeting glucose dysregulation to preserve nerve integrity in East Asian populations.

## DISCLOSURE

The authors declare that the research was conducted in the absence of any commercial or financial relationships that could be construed as a potential conflict of interest.

Approval of the research protocol: The ethics committee of the Ishibashi Clinic approved the protocol of the present research.

Informed consent: Written informed consent was obtained from all subjects based on the Declaration of Helsinki. The participants provided their written informed consent to participate in this study.

Registry and the registration no. of the study/trial: N/A.

Animal studies: N/A.

## Data Availability

The raw data supporting the conclusions of this article are available upon reasonable request. Requests for access to the data should include a clear hypothesis, detailed protocol, appropriate ethical approval, and confirmation of adherence to consent given by study participants.
